# From pipeline to network: We need to redefine scientific success

**DOI:** 10.1371/journal.pbio.3003531

**Published:** 2025-12-08

**Authors:** Alexandra A.-T. Weber

**Affiliations:** Department of Aquatic Ecology, Swiss Federal Institute of Aquatic Science and Technology (Eawag), Dübendorf, Switzerland

## Abstract

The traditional pipeline view of academia no longer reflects the reality of scientific careers. This Perspective reframes success as a network of paths recognizes excellence in its many forms, with the aim of fostering a more inclusive, resilient, and socially engaged research culture.

## Teaser

The traditional pipeline view of academia no longer reflects the reality of scientific careers. Reframing success as a network of paths recognizes excellence in its many forms, fostering a more inclusive, resilient, and socially engaged research culture.

The story of scientific success is usually told as a straight line. The academic pipeline assumes that PhD will be followed by postdoctoral position(s) and then by a permanent faculty position; the reality, however, tells a different story. Recent data from the Swiss Science Council show that only about 1% of postdoctoral researchers hold a professorship in the country four years after their PhD defense [1]. While this figure does not account for those who later secured positions or moved abroad, the overall proportion remains small, underscoring that most postdoctoral researchers do not transition to academic positions. This is not a uniquely Swiss phenomenon. A decade ago, analyses already warned of a structural imbalance between the growing number of individuals with PhDs and the limited availability of permanent faculty jobs [2]. In the United States, for example, it is estimated that only around 15% of postdoctoral researchers will eventually secure tenure-track positions [2]. What has changed since those early warnings is the urgency of the issue: the mismatch between supply and demand is now sharper, and the competition more intense.

Acknowledging this reality should not be discouraging early-career researchers (ECRs) from pursuing an academic career, least of all women, who remain underrepresented in permanent faculty positions (a pattern described historically as the “leaky pipeline” [3] and more recently as the “scissor-shaped curve” [4]). Instead, it is an invitation to awareness and empowerment. By understanding how the system works, ECRs can make informed choices, set realistic expectations, and seek out the mentorship and networks that support them. What we need is a new metaphor—not a narrow pipeline but a network of careers. In this network, scientific training connects to many domains—from academia to government and policy, patent law, industry and start-ups, education and outreach, publishing, communication, and the nonprofit sector—each offering distinct ways to apply scientific curiosity and creativity. Ultimately, success lies in finding alignment between work, values, and a sustainable, fulfilling life.

It is not hard to understand why the “pipeline” idea lingers. From the beginning, students are surrounded by academics, and professors often become the most visible role models, reflecting the well-documented influence of role models on career choices [5]. As progress is made through advanced degrees and postdoctoral positions, interactions remain almost exclusively with people who stayed in the academic system. For many, the academic environment feels intellectually stimulating and rewarding, and there is little incentive to look beyond it. Understandably, mentors advise more easily from their own experience, but this creates a form of survivorship bias [6]: ECRs mostly see the careers of those who “survived” the selection process and less often those who built equally fulfilling careers outside of academia. Without deliberate efforts to show the breadth of possibilities, many ECRs internalize the message that leaving academia is a deviation, rather than simply one of many valid outcomes of rigorous scientific training.

There is a reason many of us are drawn to academia. It offers a kind of intellectual freedom that is rare elsewhere. As such, it is not surprising that the primary career aspiration of many postdoctoral researchers is a research-focused academic position [7]. But these rewards coexist with well-known challenges [8]. Most early-career positions are temporary and require mobility [9]. For many, that mobility disrupts their social network, relationships [10], family life, and the ability to plan long-term [11]. The workload can also be intense: fieldwork is dictated by seasons, experiments can run over weekends, and grant deadlines overlap with manuscript submissions [12]. And then there is the competition. Rejection (from journals, fellowships, and grants) is part of the landscape. Unsurprisingly, the combination of insecurity, workload, and rejection takes a toll, significantly impacting the mental health and well-being of ECRs [8]. In many systems, there are few permanent positions each year, and potential institutional hiring freezes or budget restrictions make opportunities even scarcer.

Acknowledging these realities does not make academia less valuable, but it helps ECRs approach it with clear eyes and with a sense of agency in how to navigate it. For those who aspire to stay, there are ways to approach the journey with greater strategy and less uncertainty (Box 1). Still, not everyone envisions an academic future. For many, the PhD itself represents a phase of intellectual growth and skill development that opens doors to a wide range of sectors [13]. Beyond disciplinary expertise, doctoral training fosters transferable skills that are highly valued across industry, policy, and the public sector (Box 1). For instance, graduates with training in evolutionary biology [13] or biosciences [14] contribute in diverse and essential roles outside academia. Inevitably, a moment comes when many ECRs pause to ask: how long do I keep going? For some, the answer is clear. Academia still feels like the most exciting path, worth navigating uncertainty and personal costs. For others, financial stability, personal circumstances, moving fatigue, or a desire for new challenges make nonacademic careers more attractive.

**Box 1. Actionable steps for PhD holders pursuing careers within and beyond academia.**

**Within academia**

1 **Begin early**

The academic clock starts ticking with the PhD defense. Several competitive fellowships and grants have narrow eligibility windows (e.g., two or three years). Starting Grants often close eight to ten years post-PhD. Early awareness of these deadlines allows for strategic planning and avoids missed opportunities.

2 **Build comprehensive academic excellence**

Quality research remains the foundation of academic success. Yet excellence today also includes open data, reproducibility, interdisciplinary collaboration, mentoring, and societal engagement. These broader contributions are increasingly recognized in hiring and evaluation processes.

3 **Be creative and define a distinct scientific niche**

A clear scientific identity (being recognized for a particular expertise or approach) is essential for independence. It takes creativity and iteration to define a line of work that balances novelty with continuity. Successful niches grow from prior experience while pushing into new territory in ways still attractive to funders and institutions. Third-party funding, leadership in collaborations and publications without supervisors, all help consolidate this profile.

4 **Build networks and mentorship**

No one advances in isolation. Good mentors help decipher the unspoken rules of the system and provide perspective during uncertain stages. Conferences, workshops, and collaborations build visibility, while informal exchanges often spark new ideas.

5 **Develop resilience and agility**

Rejection is the rule. Every researcher has experienced grants unfunded or positions not obtained. Over time, one learns to see these moments as part of the process: opportunities to refine ideas and persist. Agility matters too, and openness to shifting focus or seizing new opportunities can turn setbacks into progress.

6 **Acknowledge factors beyond your control**

Even the best-prepared researchers depend on timing, context, and opportunity (i.e., luck). Privilege (socio-economic background, citizenship, or institutional networks) can also influence who stays longer in the academic race. Recognizing these external factors should not be discouraging, but grounding. It reminds us that careers are shaped by both agency and context, and that flexibility is often key to navigating uncertainty.

**Beyond academia**

1 **Clarify interests**

Take time to reflect on what motivates you most. Some researchers thrive in the public sector, others in policy, industry, start-ups, or nonprofit organizations. Identifying sectors that value your expertise and mapping stakeholders through institutional websites can help to reveal opportunities not always visible on traditional job boards.

2 **Learn from others**

Alumni networks and former colleagues provide valuable insights into diverse career trajectories. Their experiences illustrate how research backgrounds translate across sectors. In contexts where long-term residence is planned, learning the local language can greatly enhance integration.

3 **Recognize transferable skills**

Doctoral training develops far more than disciplinary expertise. It cultivates critical thinking, project management, data analysis, problem-solving, writing, teaching, and communication, as well as adaptability to diverse teams and cultures. Being able to articulate these skills for nonacademic audiences is crucial for successful transitions.

4 **Navigate application cultures**

Application expectations differ across sectors. Nonacademic résumés are concise and results-oriented, while cover letters emphasize achievements. Career services, professional recruiters, and peers who have transitioned can all provide valuable guidance.

5 **Strategic skill building**

When skill gaps become apparent, short courses or certificates signal adaptability and proactive growth. When possible, parts of the PhD itself could be oriented toward questions or methods relevant to industry or the public sector, helping to build a strong and versatile profile early on.

6 **Explore options**

Informal interviews and exploratory applications are valuable ways to test the waters. Applying while still in academia can refine materials, clarify competitiveness, and build confidence. Keeping such options open ensures that transitions, if they happen, are proactive rather than reactive.

The decision to stay or leave is weighty because it often feels irreversible. While there are a few successful examples of nonlinear paths—PhD, a period outside academia, and then back into academia—these remain exceptions. The competition for academic positions is already intense among those who follow the linear route. In this context, delaying the decision can carry real costs. Successive postdoctoral contracts may provide short-term stability but can narrow future options. This “postdoc trap,” staying too long without independent funding or a clear trajectory, can ultimately restrict opportunities rather than expand them. An academic leadership role is also not for everyone, and that deserves to be normalized. While the intellectual freedom largely remains, the role of a principal investigator also comes with less visible responsibilities, including budgets, administration, people management, service, and constant grant-writing. I personally find that variety stimulating, but others may find greater fulfillment in more specialized or technical roles, whether within academia or beyond.

If there is one thing to take away, it is this: success should not be defined by a single trajectory. Scientific training equips us with skills and perspectives that can flourish in many settings. Shifting from the image of a pipeline to that of a network requires effort at multiple levels. Mentors can normalize diverse outcomes and actively support transition beyond academia. Institutions can broaden success metrics to reflect the reality of where PhD graduates go. And ECRs can benefit from accurate information, supportive networks, and the confidence to define success in ways that align with their own values (Box 1).

Ultimately, success is deeply individual, and the greatest risk is letting others define it for us. By embracing a network of careers and fostering a culture that celebrates curiosity, rigor, and creativity wherever they are applied, we can build a more inclusive, resilient, and sustainable scientific community: one where the true measure of success is a life and career that feel meaningful and fulfilling.

## Abbreviation:

ECR: early-career researcher
